# Supplementary material for: Mannose ameliorates experimental colitis by protecting intestinal barrier integrity
Source: Nat Commun. 2022 Aug 16;13:4804. doi: 10.1038/s41467-022-32505-8 (PMC9381535; doi:10.1038/s41467-022-32505-8)
Supplement: Supplementary file 1 — Supplementary information [file 41467_2022_32505_MOESM1_ESM.pdf]

Mannose ameliorates experimental colitis by protecting intestinal barrier integrity

Supplementary information

Supplementary Figure 1

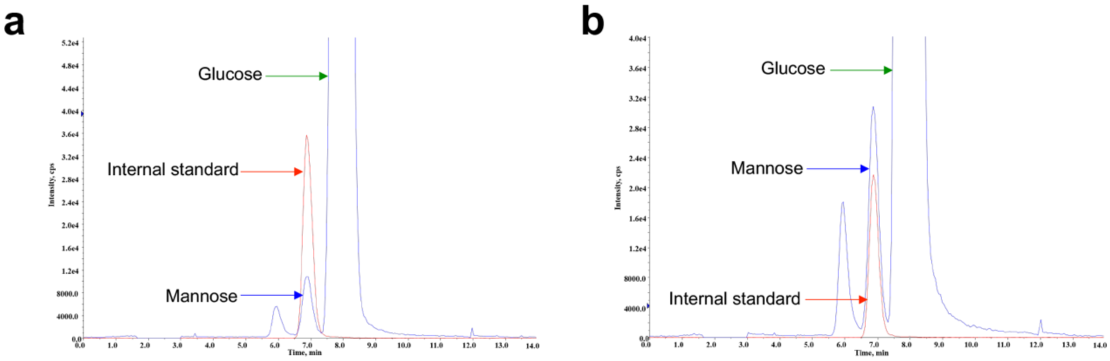

Figure S1. The chromatography of mannose in biological samples.

Serum samples were mixed with methanol-acetonitrile-water (v:v:v, 2:2:1) mixed solvent containing 5 μg/ml D-mannose-1-^{13}C IS. After centrifugation, an aliquot (10 μl) of the supernatant was taken for LC-MS/MS analysis. Representative chromatogram of human plasma (blue arrow) (a) and mouse serum (blue arrow) (b) sample spiked with internal standard (mannose-1-^{13}C, red arrow).

Supplementary Figure 2

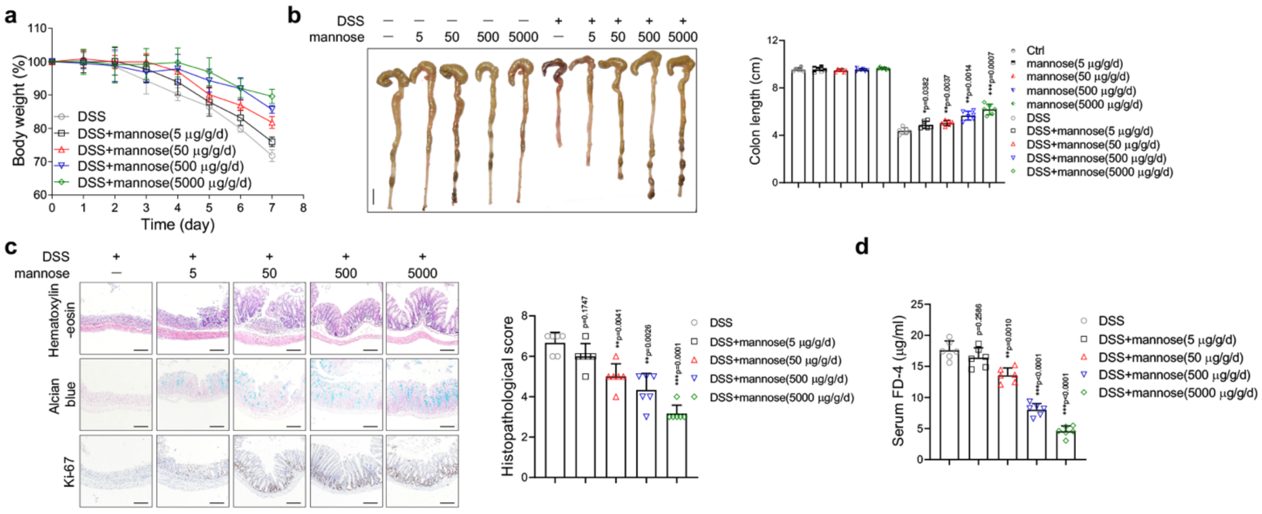

Figure S2. Mannose attenuates DSS-induced colitis in a dose-dependent manner.

Mice (male, n=6 per group) were treated with 3.0% DSS in the presence or absence of the indicated doses of mannose for 7 consecutive days. (a) The body weight changes during the experiments were measured daily. (b) Colon tissues were isolated on day 7 of the experiment, and the colon length was recorded (Scale bar=1 cm). (c) The histological analysis of mouse colon tissue taken on the last day of the experiment was performed by H&E, alcian blue, and Ki67 staining. Scale bar=100 μm. Histological scores of the DSS-induced colitis were evaluated (9 slides/sample) (d) Intestinal permeability was determined by the serum FD-4 concentration on day 7 after the DSS challenge compared with the group treated with DSS alone. Data from one representative experiment of three independent experiments are presented, $*p<0.05$, $**p<0.01$, $***p<0.001$. Data were analyzed by an unpaired Student's t-test(b-d) and shown as means ± SD. Data from one representative experiment of three independent experiments are presented. Source data are provided as a Source Data file.

Supplementary Figure 3

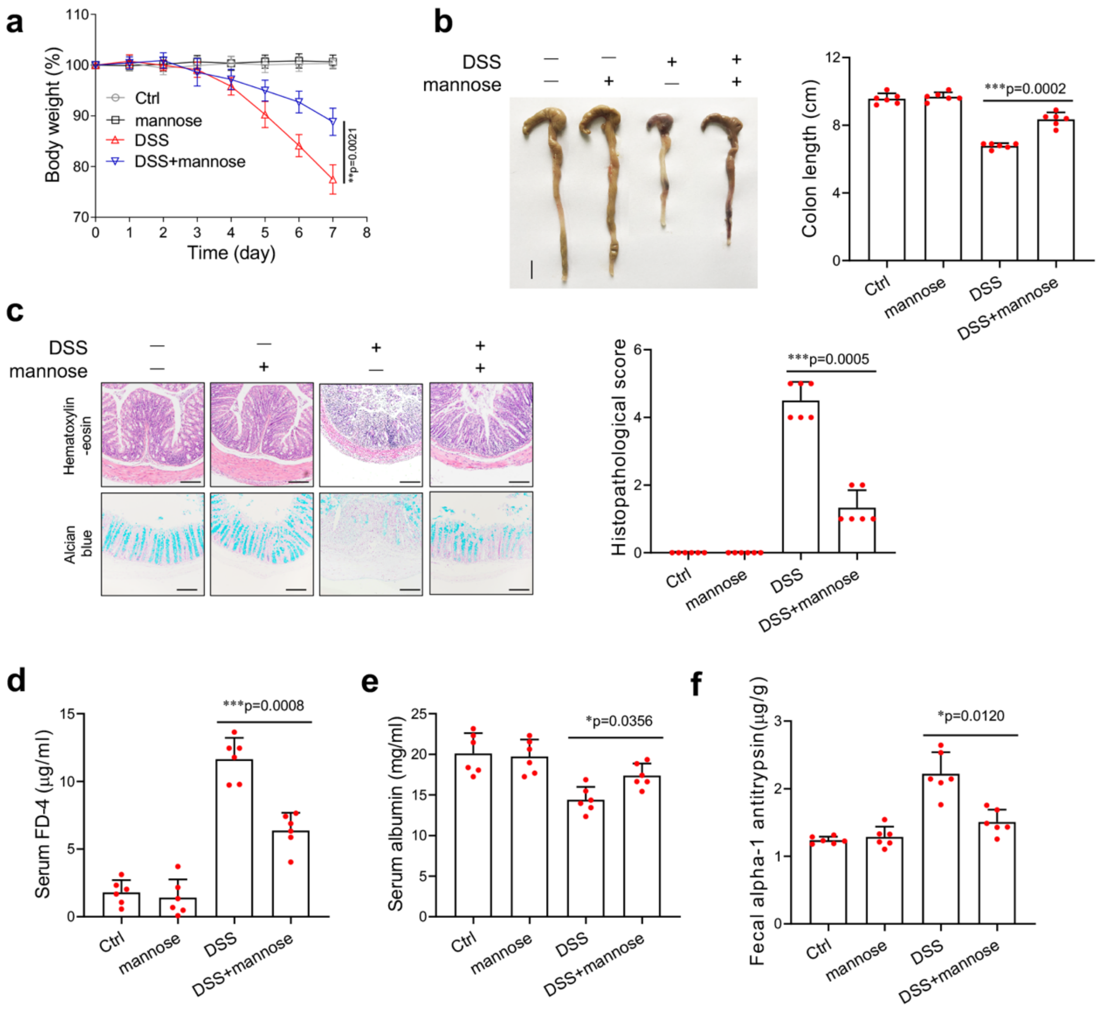

Figure S3. Mannose administration ameliorates DSS-induced colitis in female mice.

Female mice (n=6 per group) were treated with 3.0% DSS in the presence or absence of mannose (500 µg/g/d) for 7 consecutive days. (a) The body weight changes during the experiments were monitored (b) Colon tissues were isolated on the last day of the experiment. A representative photograph of colon tissue from each group is provided, and the colon length was recorded. (c) The histological analysis of mouse colon tissue taken on the last day of the experiment was performed by hematoxylin and eosin (H&E), and alcian blue. (Scale bar=100 µm). Histological scores of the DSS-induced colitis were evaluated (9 slides/sample). (d-f) Intestinal permeability was determined by serum FD-4 concentration

(d), the albumin level of serum (e) and fecal α1-antitrypsin (f) on day 7 after DSS challenge compared with the group treated with DSS alone. Data from one representative experiment of three independent experiments are presented. **$p<0.01$, ***$p<0.001$. Data are means ± SD. Two-side, unpaired t-test for a–f.

Supplementary Figure 4

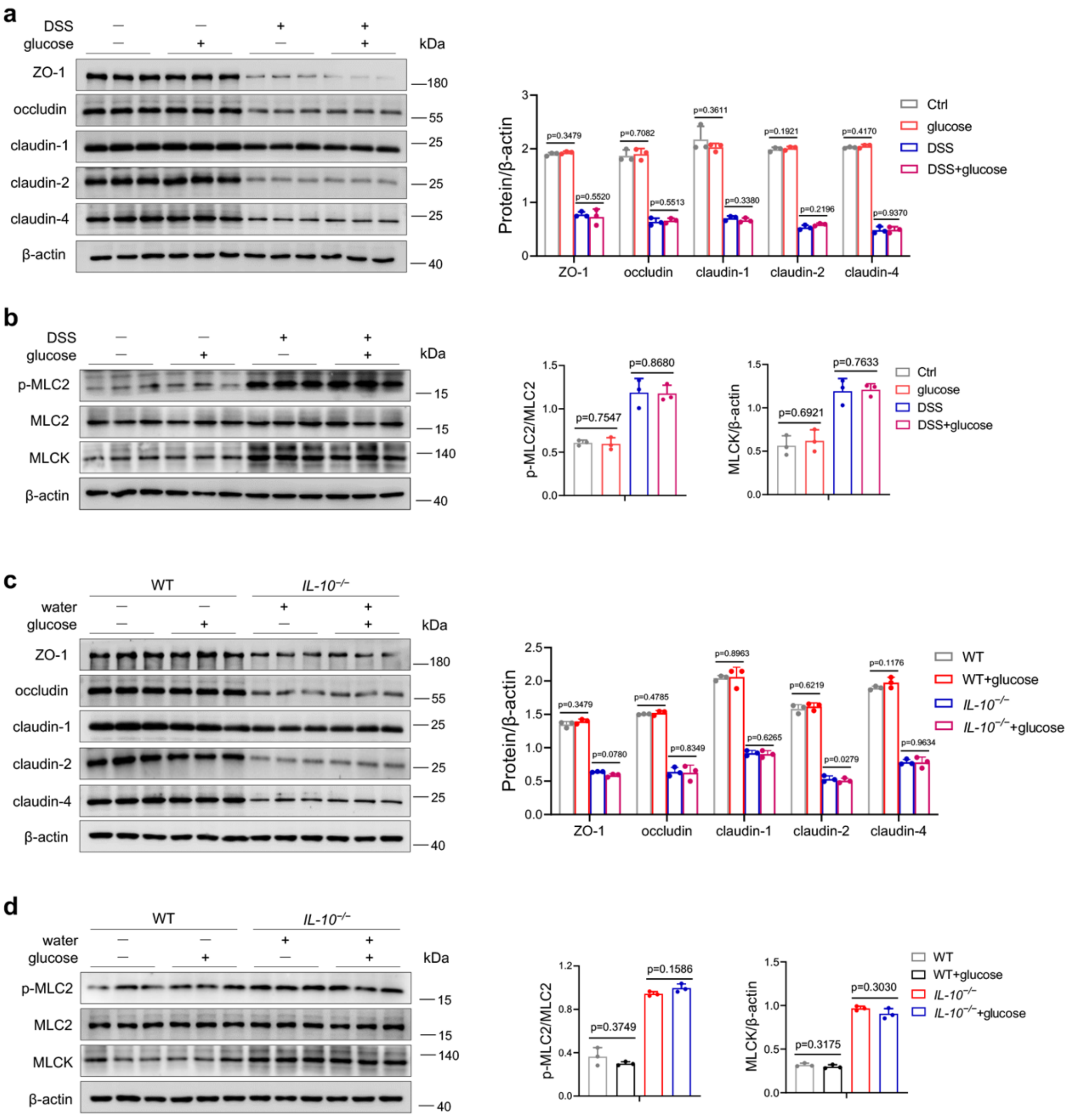

Figure S4. Glucose has no effect on tight junction properties in both DSS-induced colitis and *IL-10*$^{-/-}$ spontaneous colitis.

Mice (male, n=6 per group) were treated with 3.0% DSS in the presence or absence of the indicated doses of glucose for 7 consecutive days. (a, b) The expression of tight junction proteins, MLC2, phospho-MLC2, and MLCK in the mouse colon was determined by western blot analysis (c, d) The *IL-10*$^{-/-}$ mice (15 weeks old, n=6 per group) were fed 1.0% glucose for 4 weeks. The expression of

tight junction proteins, MLC2, phospho-MLC2, and MLCK in the mouse colon was determined by western blot analysis. Data were analyzed by an unpaired Student's t-test(a-f) and shown as means ± SD. Data from one representative experiment of three independent experiments are presented. Source data are provided as a Source Data file.

Supplementary Figure 5

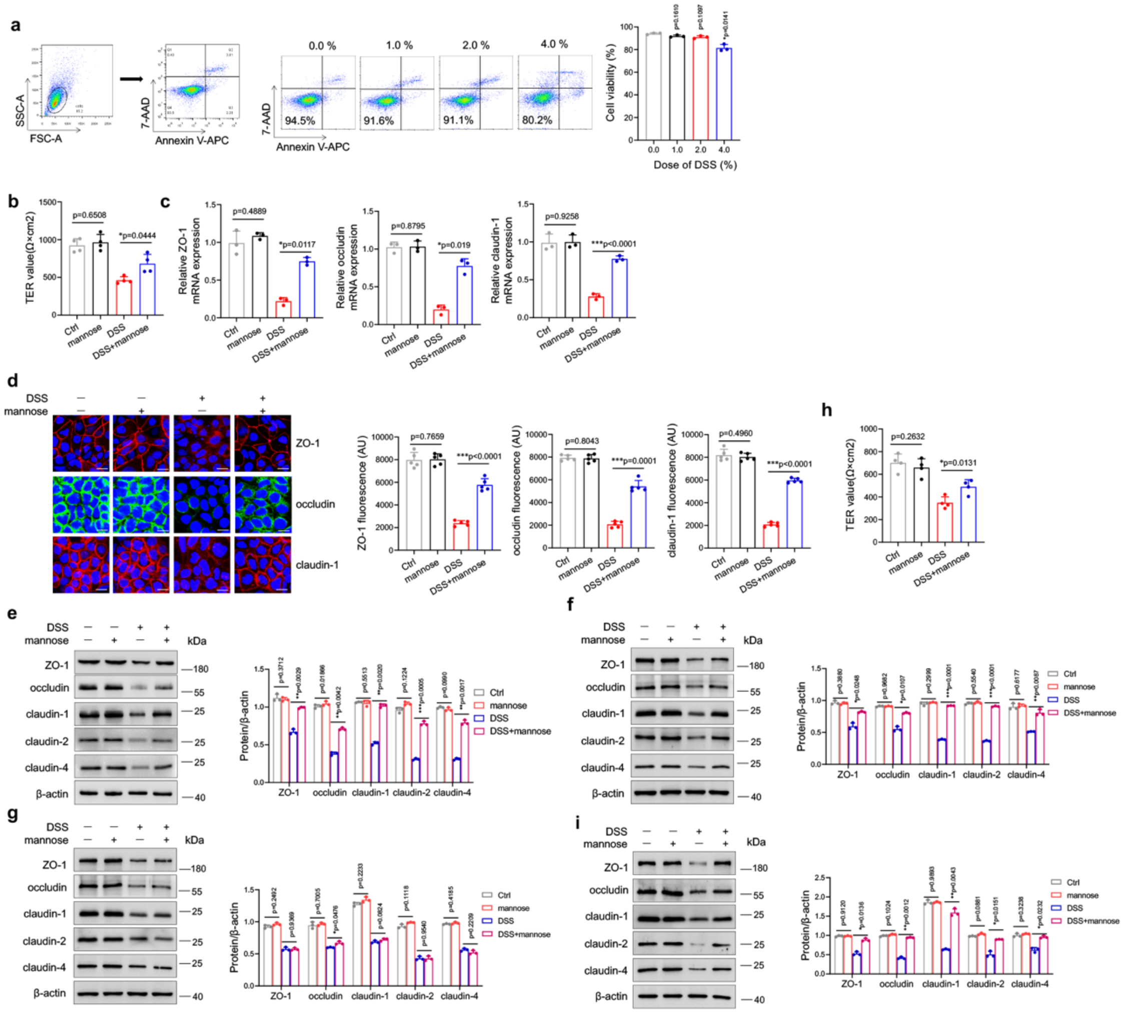

Figure S5. Mannose prevents the DSS-induced loss of epithelial cell tight junction.

(a) NCM460 cells were stimulated with the indicated concentration of DSS for 24 hours. The cell viability was determined by flow cytometry. (b-e) NCM460 cells were treated with 2.0% DSS in the presence or absence of mannose (25 mM) for 24 hours. The TER value of the NCM460 monolayers was measured (b). The mRNA levels of ZO-1, occludin, and claudin-1 in cells were examined by qRT-PCR analysis (c) and the protein levels of tight junction proteins were determined by immunofluorescence staining (d). and western blot analysis (Scale bar=20 µm). (e). (f) NCM460 cells were treated with DSS for 12 hours, followed by incubated with mannose for another 12 hours. The

expression of tight junction proteins was examined by western blot analysis (g) NCM460 cells were pretreated with mannose for 12 hours, followed by stimulation with DSS for another 12 hours. The expression of tight junction proteins was examined by western blot analysis. (h, i) Primary colonic epithelial cells isolated from C57BL/6J mice were treated with 2.0% DSS in the presence or absence of mannose (5 mM) for 24 hours. The TER value of primary colon epithelial cells was also detected (h). The levels of tight junction proteins in the primary colonic epithelial cells were measured by western blotting (i). Data from one representative experiment of three independent experiments are presented. *$p<0.05$, **$p<0.01$, ***$p<0.001$. Data were analyzed by an unpaired Student's t-test(a-i) and shown as means ± SD. Source data are provided as a Source Data file.

Supplementary Figure 6

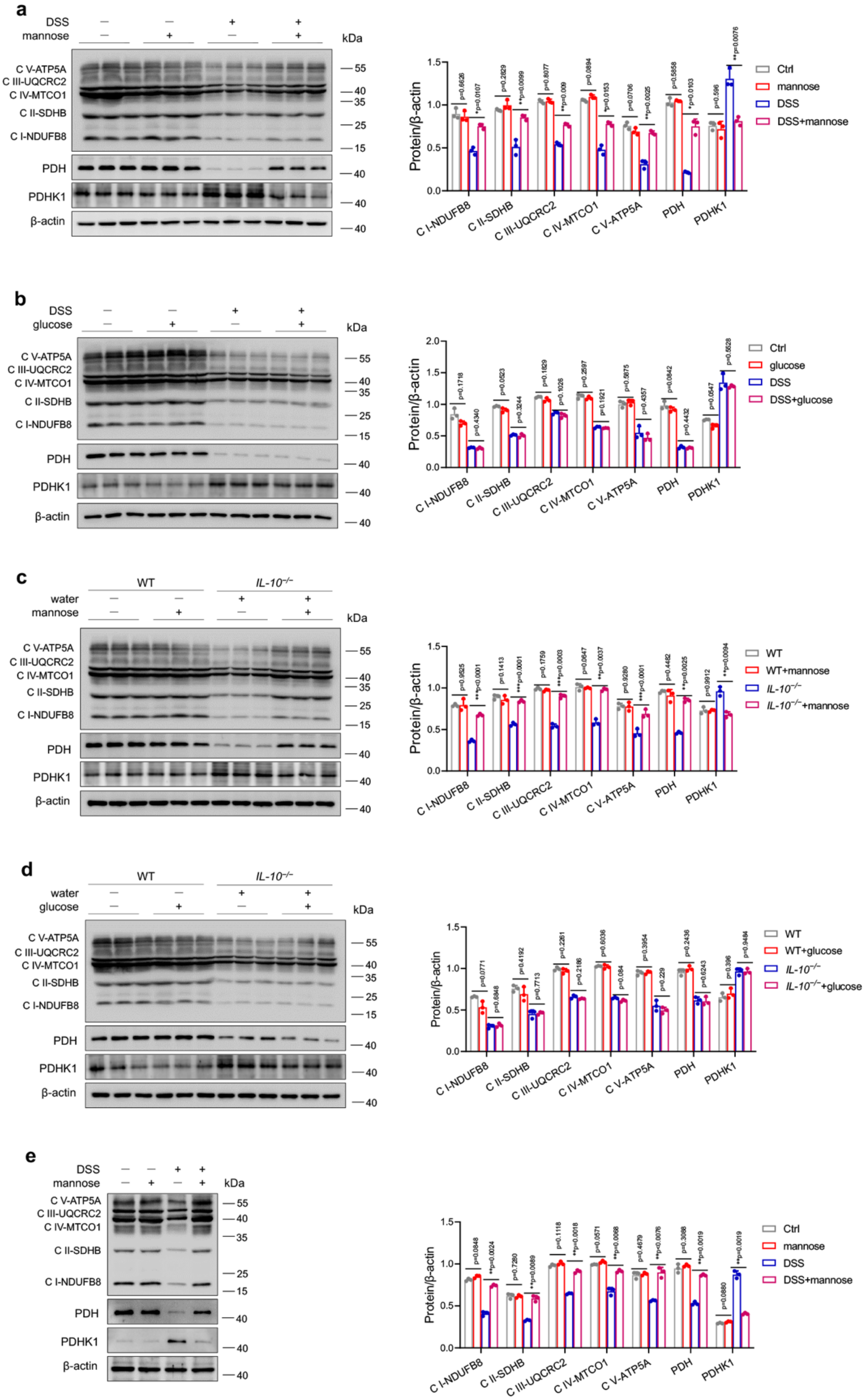

Figure S6. Mannose rescues the colitis-induced alterations of oxidative phosphorylation in colonic tissue.

(a, b) C57BL/6J mice (male, n=6 per group) were treated with 3.0% DSS in the presence or absence of mannose or glucose (500 μg/g/d) for 7 consecutive days. Colon tissue was isolated on the last day of the experiment. The levels of OXPHOS, PDH, and PDHK1 in the colon tissue were evaluated by immunoblotting analysis. (c, d) *IL-10−/−* mice (15 weeks old, n=6 per group) were fed 1.0% mannose or glucose for 4 weeks. The expression of OXPHOS, PDH, and PDHK1 in the mice colon tissue was determined on the last day of the experiment by western blot analysis (e) Primary colon epithelial cells isolated from mice were treated with 2.0% DSS in the presence or absence of mannose (5 mM) for 24 hours. The expression of OXPHOS, PDH, and PDHK1 in the cells was measured by western blotting Data from one representative experiment of three independent experiments are presented. *$p<0.05$, **$p<0.01$, ***$p<0.001$. Data are means ± SD. Two-side, unpaired t-test for a–e.

Supplementary Figure 7

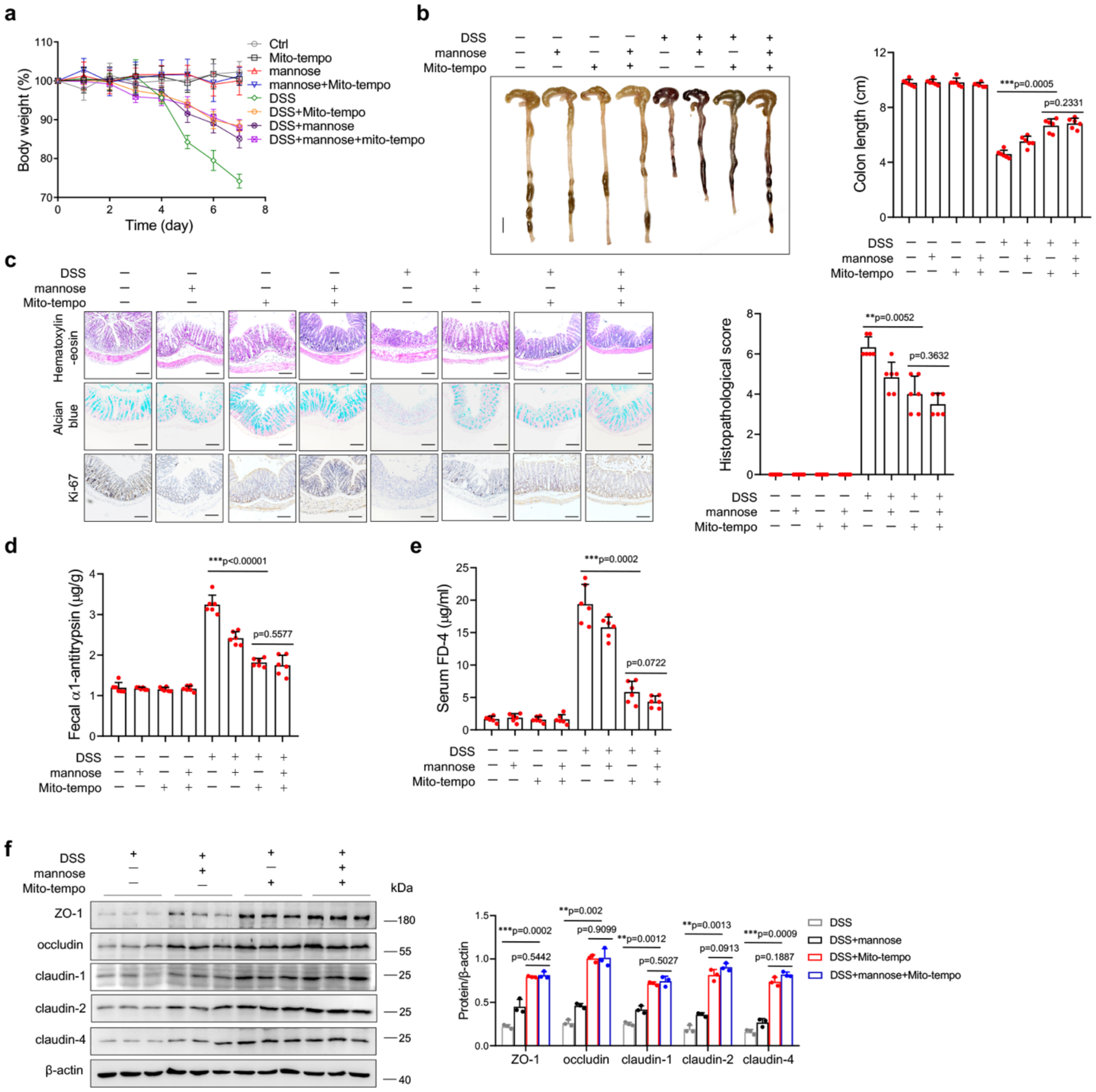

Figure S7. Mannose ameliorates DSS-induced colitis by regulating mitochondrial function.

The mice (male, n=6, per group) were treated with 3.0% DSS and mannose (500 μg/g/d) in the presence of mito-tempo (2 mg/kg) for 7 consecutive days. (a) The body weight changes during the experiments were recorded. (b) Colon tissues were isolated on the last day of the experiment. A representative photograph of the colon tissue from each group is provided, and the colon length was recorded (Scale bar=1 cm). (c) Histological analysis of mouse colon tissue taken on the last day of the experiment was

performed by H&E, alcian blue, and Ki67 staining. Scale bar=100 μm. Histological scores of the DSS-induced colitis were evaluated (9 slides/sample). Intestinal permeability was determined by the fecal α1-antitrypsin level (d) and serum FD-4 concentration (e) on day 7 after the DSS challenge. (f) The levels of tight junction proteins in the colon tissue were detected by western blotting. Data from one representative experiment of three independent experiments are presented. $^{*}p<0.05$, $^{**}p<0.01$, $^{***}p<0.001$. Data are means ± SD. Two-side, unpaired t-test for b–f. Source data are provided as a Source Data file.

Supplementary Figure 8

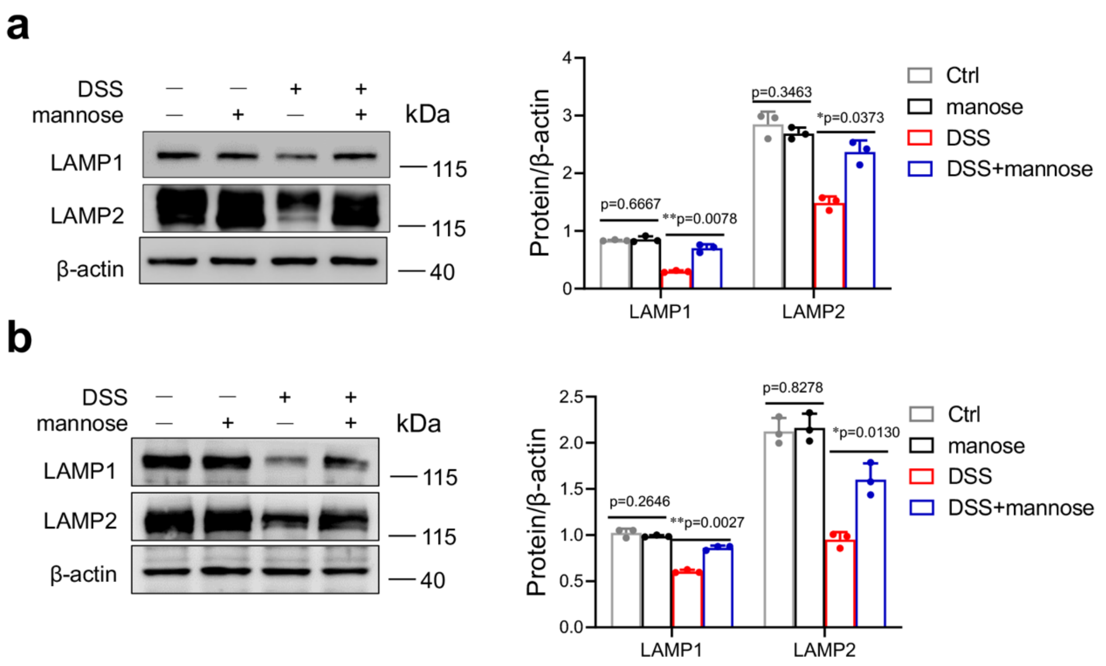

Figure S8. Mannose rescues the DSS-induced alterations of lysosomal associated membrane protein in epithelial cells.

(a) NCM460 cells were treated with 2.0% DSS in the presence or absence of mannose (25 mM) for 24 hours. The levels of LAMP 1 and LAMP 2 in the cells were examined by western blot analysis (b) Primary colonic epithelial cells isolated from mice were treated with 2.0% DSS in the presence or absence of mannose (5 mM) for 24 hours. The expression levels of LAMP1 and LAMP2 in the primary colonic epithelial cells were determined by western blotting. Data from one representative experiment of three independent experiments are presented. *p<0.05, **p<0.01. Data were analyzed by an unpaired Student's t-test(a-b) and shown as means ± SD. Source data are provided as a Source Data file.

Supplementary Figure 9

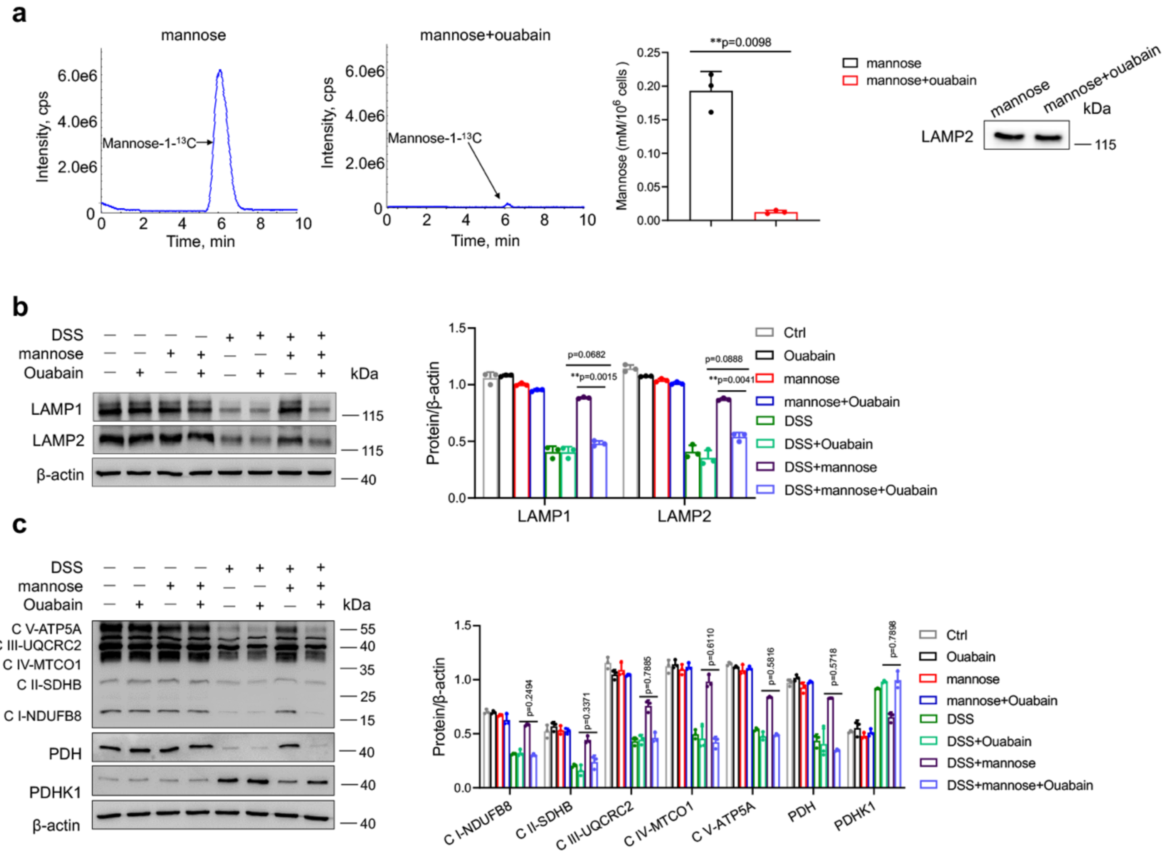

Figure S9. The mannose transporter is involved in mannose-mediated protection in DSS-treated NCM460 cells.

(a) NCM460 cells were incubated with 25mM mannose-1-^{13}C for 24 hours in the presence of ouabain. Subsequently, the lysosomal fraction was isolated from the mannose-treated cells and the presence of mannose in the lysosomal fraction was determined by LC-MS/MS analysis. (b, c) NCM460 cells were cultured with DSS and mannose for 24 hours in the presence or absence of ouabain. The levels of LAMP1, LAMP2, and OXPHOS proteins were determined by western blot analysis. Data from one representative experiment of three independent experiments are presented. *p<0.05, **p<0.01, ***p<0.001. Data are means ± SD. Two-side, unpaired t-test for a-c. Source data are provided as a Source Data file.

Supplementary Figure 10

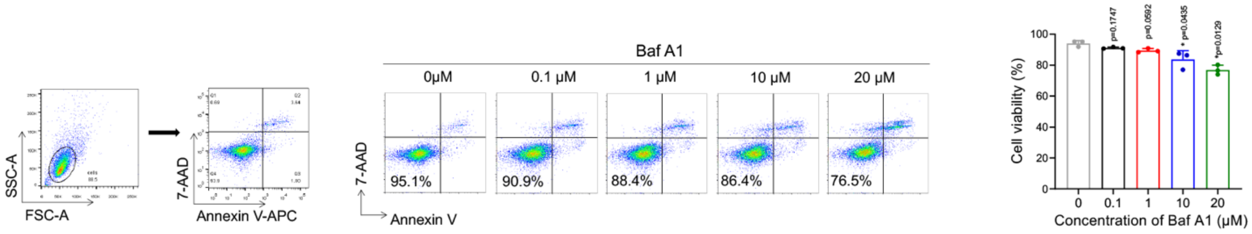

Figure S10. The effects of bafilomycin A 1 on cell viability of NCM460 cells.

NCM460 cells were stimulated with the indicated concentration of bafilomycin A 1 for 24 hours. The cell viability was determined by flow cytometry. Data from one representative experiment of three independent experiments are presented. *$p<0.05$. Data are means ± SD. Two-side, unpaired t-test. Source data are provided as a Source Data file.

Supplementary Figure 11

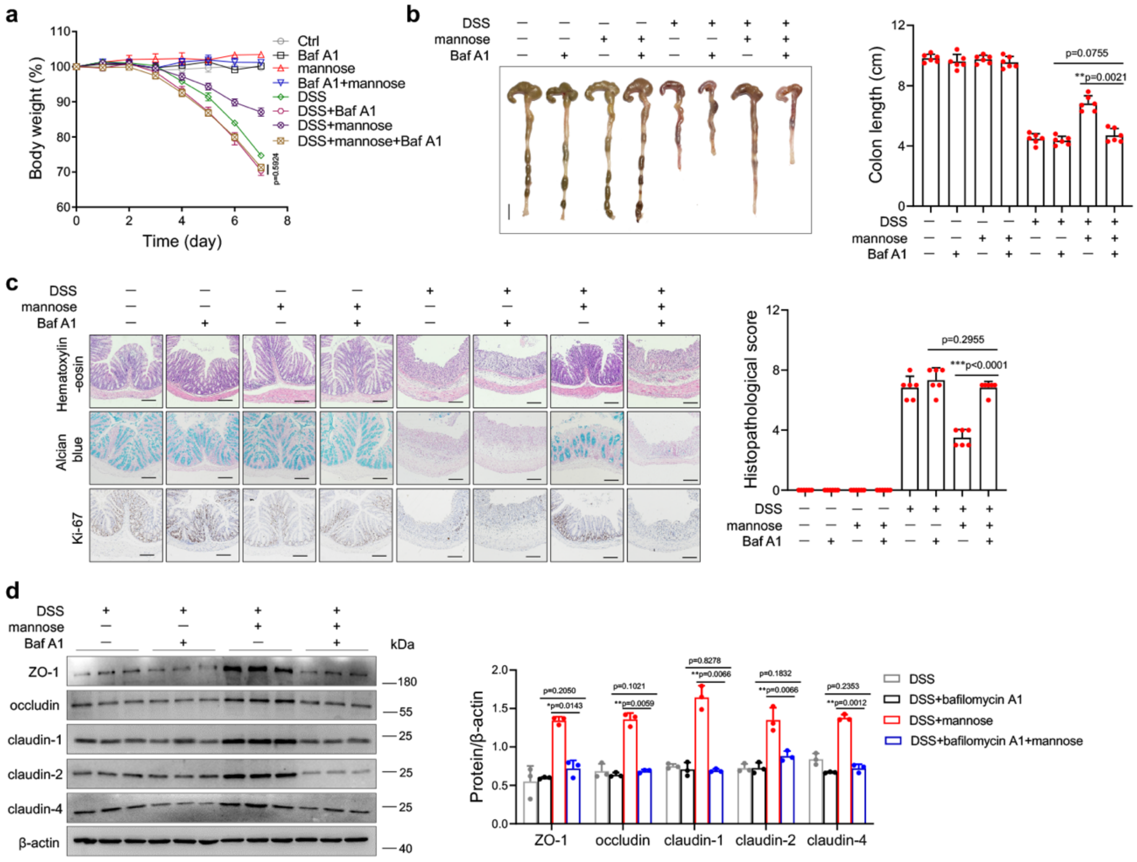

Figure S11. Mannose ameliorates DSS-induced colitis by regulating lysosomal function.

The mice (male, n=6 per group) were treated with 3.0% DSS and mannose (500 μg/g/d) in the presence of bafilomycin A 1 (2 mg/kg) for 7 consecutive days. (a) The body weight changes during the experiments were recorded. (b) Colon tissues were isolated on the last day of the experiment. A representative photograph of the colon tissue from each group is provided, and the colon length was recorded. (Scale bar=1 cm). (c) Histological analysis of mouse colon tissue taken on the last day of the experiment was performed by H&E, alcian blue, and Ki67 staining. Scale bar=100 μm. Histological scores of the DSS-induced colitis were evaluated (9 slides/sample). (d) The levels of tight junction proteins in the colon tissues were detected by western blotting. Data from one representative

experiment of three independent experiments are presented. *$p<0.05$, **$p<0.01$, ***$p<0.001$. Data are means ± SD. Two-side, unpaired t-test for a-d. Source data are provided as a Source Data file.

146 **Supplementary Figure 12**

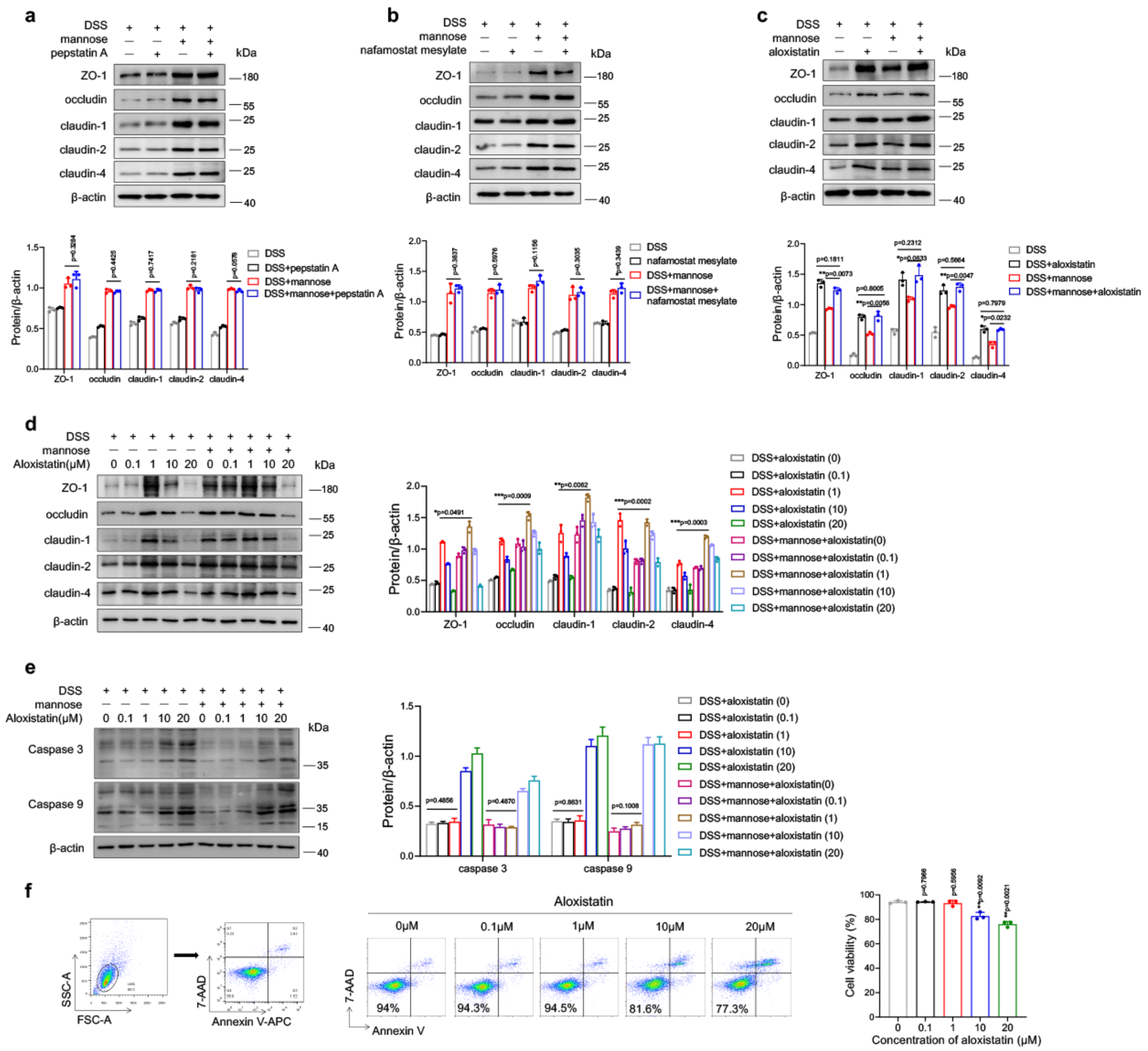

Figure S12. Cysteine protease activity is involved in the mannose-mediated protection of tight junctions in DSS-treated NCM460 cells.

(a-c) NCM460 cells were treated respectively with 10 µM of pepstatin A (a), 5 µM of nafamostat mesylate(b), or 1 µM aloxistatin (c), followed by DSS and mannose stimulation. The levels of ZO-1, occludin, and claudin-1, 2, 4 in the cells were determined by western blot analysis. (d, e) DSS-treated NCM460 cells were stimulated with different concentrations of aloxistatin in the presence or absence of mannose. The levels of tight junction in the cells were examined by immunoblotting analysis (e). (f) NCM460 cells were stimulated with indicated concentration of aloxistatin for 24 hours. The cell

viability was determined by flow cytometry. Data from one representative experiment of three independent experiments are presented. *$p<0.05$, **$p<0.01$, ***$p<0.001$. Data are means ± SD. Two-side, unpaired t-test for a-f. Source data are provided as a Source Data file.

Supplementary Figure 13

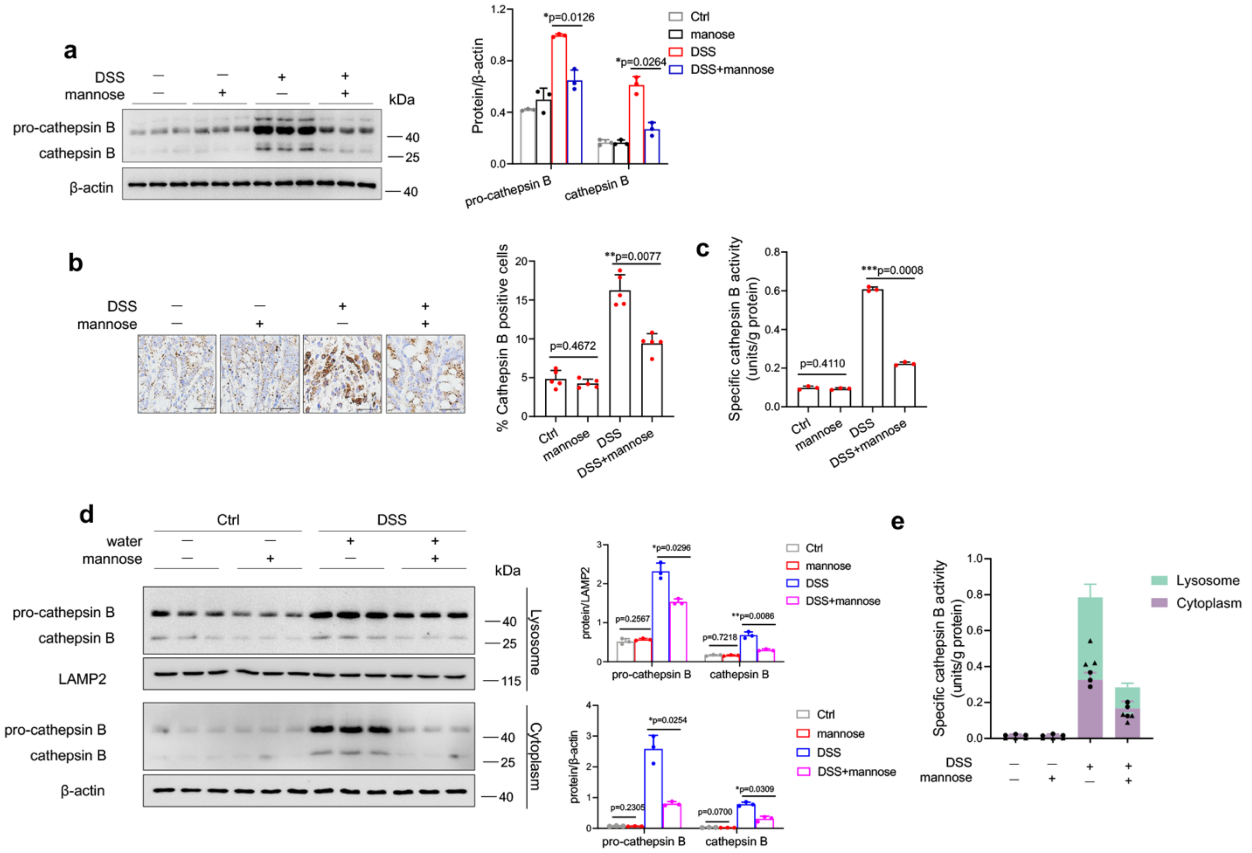

Supplementary Figure 13. Mannose treatment decreases the expression of cathepsin B in the colon tissues from the DSS-induced colitis model mice

(a-c) Mice (male, n=6 per group) were treated with 3.0% DSS in the presence or absence of mannose (500 µg/g/d) for 7 consecutive days. The protein level of activated cathepsin B in the colon tissues was evaluated by western blotting (a) and immunohistochemical staining (Scale bar=50 µm) (b). The activity of cathepsin B in the colon homogenate was assessed (c). The primary colonic epithelial cells were isolated on the last day of the experiment. The expression level (d) and the activity (e) of cathepsin B in the lysosome and other remaining cytoplasm of the colonic epithelial cells were evaluated. Data from one representative experiment of three independent experiments are presented. *p<0.05, **p<0.01. Data are means ± SD. Two-side, unpaired t-test for a-f. Source data are provided as a Source Data file.

173 **Supplementary Figure 14**

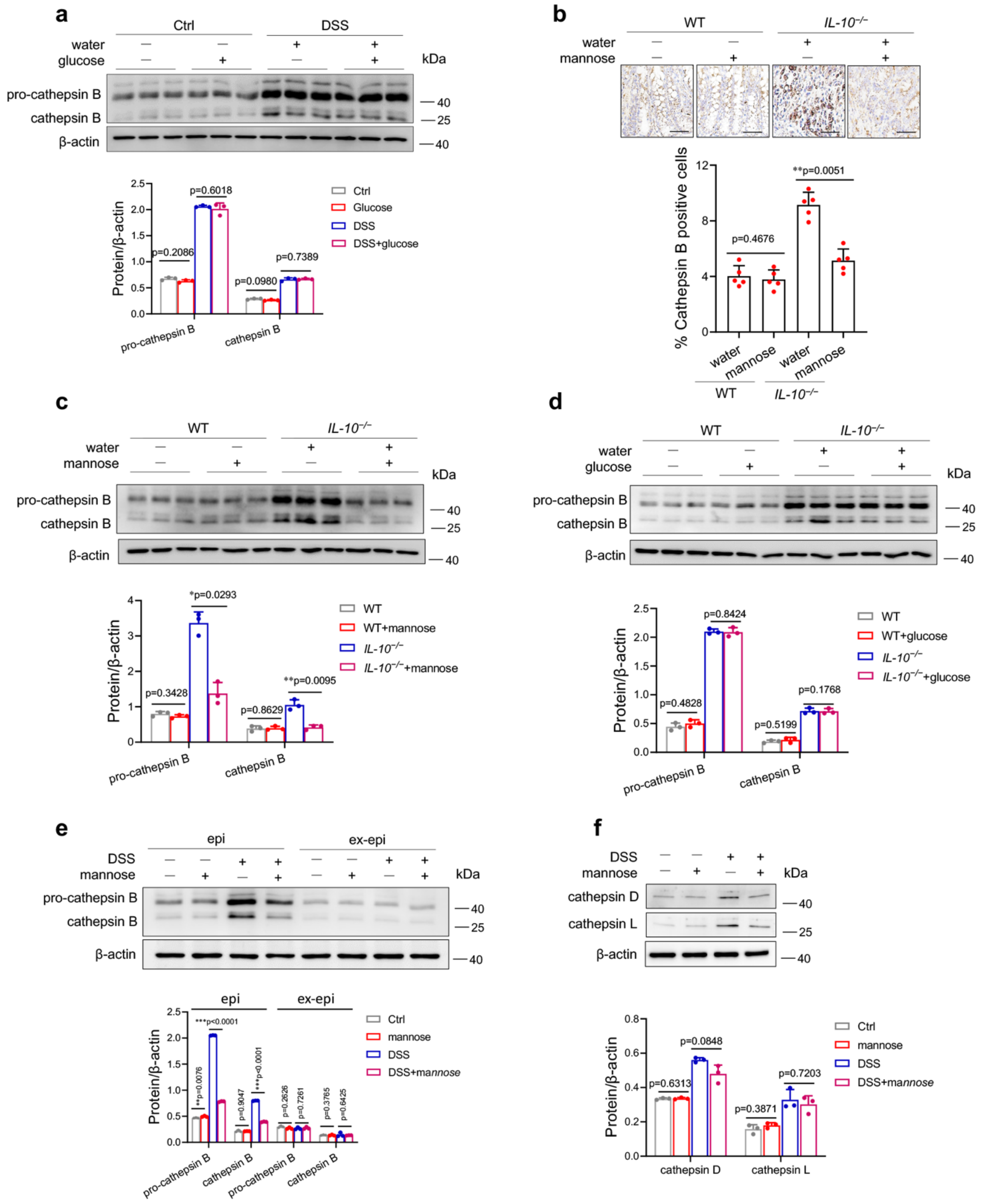

174

175 **Figure S14. Mannose treatment limits cathepsin B release in the colon in colitis mice.**

(a) Mice (male, n=6 per group) were treated with 3.0% DSS in the presence or absence of glucose (500 μg/g/d) for 7 consecutive days. The protein level of mature cathepsin B in the colon tissues was evaluated by western blotting. (b, c) *IL-10*$^{-/-}$ mice (15 weeks old, n=6 per group) were fed 1.0% mannose for 4 weeks. The expression of cathepsin B in the colon was determined on the last day of the experiment by immunohistochemical staining (b) and western blot analysis (Scale bar=50 μm) (c). (d) *IL-10*$^{-/-}$ mice (15 weeks old, n=6 per group) were fed 1.0% glucose for 4 weeks. The expression of cathepsin B in the colon was determined on the last day of the experiment by western blot analysis. (e, f) C57BL/6J mice were treated with 3.0% DSS in the presence or absence of mannose (500 μg/g/d) for 7 consecutive days. Colon epithelial cells were isolated on the last day of the experiment, and the expression levels of cathepsin B in the colonic epithelial cells and other remaining parts were evaluated by immunoblotting analysis. (f) The colon tissue was isolated on the last day of the experiment. Then the levels of cathepsin L and cathepsin D in the colon tissues were evaluated by immunoblotting analysis. Data from one representative experiment of three independent experiments are presented. *p<0.05, **p<0.01, ***p<0.001. Data are means ± SD. Two-side, unpaired t-test for a-f. Source data are provided as a Source Data file.

Supplementary Figure 15

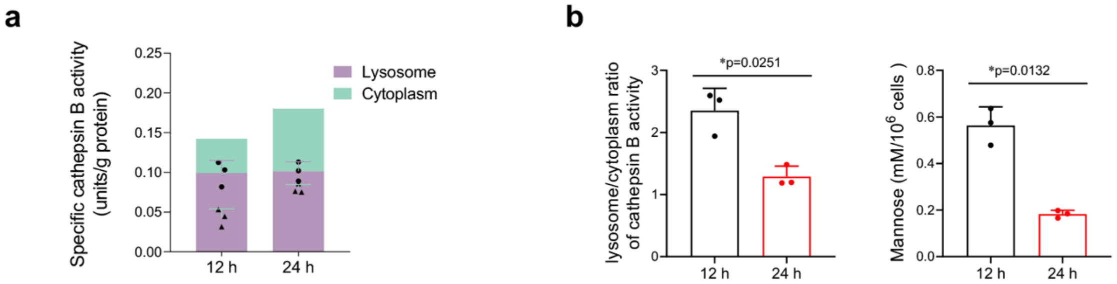

Figure S15. Mannose-mediated inhibitory effect of cathepsin B release from lysosome is associated with mannose level in the lysosome.

(a, b) NCM460 cells were stimulated with 2.0% DSS in the presence of ^{13}C-labeled mannose (25mM) for 12 and 24 hours. Then, the cells were collected and lyzed for further analysis. (a) The activities of cathepsin B in the lysosome and cytoplasm of NCM460 cells were assessed, and the lysosome/cytoplasm ratio of cathepsin B activity in the cells was calculated(b) The amount of ^{13}C-labeled mannose in the cell lysosome was determined by LC-MS/MS analysis. Data from one representative experiment of three independent experiments are presented. *$p<0.05$. Data are means ± SD. Two-side, unpaired t-test for a-b. Source data are provided as a Source Data file.

Supplemental Table 1. Characteristics of patients with IBD and HCs

	Controls	UC	CD
**Numbers**	30	35	9
**Mean age, y (range)**	36.21 (19-47)	32.6 (19-47)	28 (13-42)
**Gender (no. of male patients /no. of female patients)**	17/13	20/15	6/3
**Mean disease duration, y (range)**	—	4.5(1-14)	4.8 (1-13)
**Symptoms**	—		
		Blood in stool: 16	Blood in stool: 1
		Abdominal pain: 13	Abdominal pain: 3
		Diarrhea: 11	Diarrhea: 4
		Weight loss: 6	Weight loss: 3
		Other (eg, anemia, vomiting, perianal problems): 3	Other (eg, anemia, fever, fatigue, perianal problems): 2
**Disease extent**	—	Proctitis: 5	Ileal involvement: 1
		Left-sided ulcerative colitis: 14	Colonic involvement: 4
		Pancolitis: 16	Ileocolonic involvement: 4
**Active/remission**	—	24/11	7/2
**Smoking**			
Never smoking	30	30	8
Quit smoking	0	2	0
On smoking	0	3	1
**Extraintestinal manifestations**	—		
**Clinical severity**			
Mild		14	3
Moderate		12	4
Severe		5	1
Remission		4	1
**Medication**	—		
Salazosulfapyridine or mesalazine		4	0
Corticosteroids		2	0
Immunosuppressants		26	7
Infliximab		3	2
**Behaviour**	—		
Inflammatory		18	5
Fistulizing		12	4
Stricturing		4	0
Perianal Involvement		1	0
