# Supplementary material for: Mannose ameliorates experimental colitis by protecting intestinal barrier integrity
Source: Nat Commun. 2022 Aug 16;13:4804. doi: 10.1038/s41467-022-32505-8 (PMC9381535; doi:10.1038/s41467-022-32505-8)
Supplement: Supplementary file 2 — Reporting Summary [file 41467_2022_32505_MOESM2_ESM.pdf]

nature research

Corresponding author(s): Qingqing Wang; Zhengliang Chen; Daming Zuo

Last updated by author(s): Jun 27, 2022

Reporting Summary

Nature Research wishes to improve the reproducibility of the work that we publish. This form provides structure for consistency and transparency in reporting. For further information on Nature Research policies, see Authors & Referees and the Editorial Policy Checklist .

Statistics

For all statistical analyses, confirm that the following items are present in the figure legend, table legend, main text, or Methods section.

n/a	Confirmed	
☐	☒	The exact sample size (n) for each experimental group/condition, given as a discrete number and unit of measurement
☐	☒	A statement on whether measurements were taken from distinct samples or whether the same sample was measured repeatedly
☐	☒	The statistical test(s) used AND whether they are one- or two-sided<br>*Only common tests should be described solely by name; describe more complex techniques in the Methods section.*
☒	☐	A description of all covariates tested
☐	☒	A description of any assumptions or corrections, such as tests of normality and adjustment for multiple comparisons
☐	☒	A full description of the statistical parameters including central tendency (e.g. means) or other basic estimates (e.g. regression coefficient) AND variation (e.g. standard deviation) or associated estimates of uncertainty (e.g. confidence intervals)
☐	☒	For null hypothesis testing, the test statistic (e.g. F, t, r) with confidence intervals, effect sizes, degrees of freedom and P value noted<br>*Give P values as exact values whenever suitable.*
☒	☐	For Bayesian analysis, information on the choice of priors and Markov chain Monte Carlo settings
☒	☐	For hierarchical and complex designs, identification of the appropriate level for tests and full reporting of outcomes
☒	☐	Estimates of effect sizes (e.g. Cohen's d, Pearson's r), indicating how they were calculated

Our web collection on statistics for biologists contains articles on many of the points above.

Software and code

Policy information about availability of computer code

Data collection	Images: Nikon A1R scanning laser confocal microscope<br>Western blotting: Tanon Imager<br>Flow cytometry: Dakewe EXFLOW<br>Microsoft Excel 2013
Data analysis	Graphpad Prism 6, SPSS version 23, Microsoft Excel 2013,Image J v1.8.0, FlowJo v 7.6.1

For manuscripts utilizing custom algorithms or software that are central to the research but not yet described in published literature, software must be made available to editors/reviewers. We strongly encourage code deposition in a community repository (e.g. GitHub). See the Nature Research guidelines for submitting code & software for further information.

Data

Policy information about availability of data

All manuscripts must include a data availability statement. This statement should provide the following information, where applicable:
- Accession codes, unique identifiers, or web links for publicly available datasets
- A list of figures that have associated raw data
- A description of any restrictions on data availability

The authors declare that all data supporting the findings of this study are available within the article and its Supplementary Information files or are available from the authors on request. The source data underlying Figs 1,2,3,4,5,6,7 and Supplementary Figs 1-15 are provided as a Source Data file.

Field-specific reporting

Please select the one below that is the best fit for your research. If you are not sure, read the appropriate sections before making your selection.

- [x] Life sciences
- [ ] Behavioural & social sciences
- [ ] Ecological, evolutionary & environmental sciences

For a reference copy of the document with all sections, see nature.com/documents/nr-reporting-summary-flat.pdf

Life sciences study design

All studies must disclose on these points even when the disclosure is negative.

Sample size	In Vivo experiments were performed using a sample size of n=6-10 mice per arm unless noted otherwise. Power analysis was used to decide on the number of mice per group. For immunofluorescence analysis, multiple fields of view per slide were quantified, and exact numbers are specified in the methods section of the paper. In most experiments, at least three biological repeats were performed. The number of patients included in the study was determined by the availability of patients' specimens.
Data exclusions	No data was excluded for this study.
Replication	Unless specified, data were representative of three independent experiments. All the experiments and analyses shown could be successfully and reliably replicated and reproduced.
Randomization	Before the experiments, all the samples were grouped randomly for each participant, and repeat these results at different time.
Blinding	All the experiments were double blinded. One of the participant prepare samples, another one perform the experiments, finally all participants analyzed the results together.

Reporting for specific materials, systems and methods

We require information from authors about some types of materials, experimental systems and methods used in many studies. Here, indicate whether each material, system or method listed is relevant to your study. If you are not sure if a list item applies to your research, read the appropriate section before selecting a response.

Materials & experimental systems

n/a	Involved in the study	
[ ]	[x]	Antibodies
[ ]	[x]	Eukaryotic cell lines
[x]	[ ]	Palaeontology
[ ]	[x]	Animals and other organisms
[ ]	[x]	Human research participants
[x]	[ ]	Clinical data

Methods

n/a	Involved in the study	
[x]	[ ]	ChIP-seq
[ ]	[x]	Flow cytometry
[x]	[ ]	MRI-based neuroimaging

Antibodies

Antibodies used

β-actin (Rabbit monoclonal [13E5], 1:1000 for immunoblotting, Cell Signaling Technology, 4970)
ZO-1 (Rabbit monoclonal [13E5], 1:200 for immunostaining, 1:2000 for immunoblotting, Thermo Fisher,61-7300, SH252320)
Claudin1(Rabbit polyclonal, 1:200 for immunostaining,1:1000 for immunoblotting, Proteintech, 13050-1-AP)
p-MLC2 (Rabbit monoclonal, 1:1000 for immunoblotting, Cell Signaling Technology, 3674)
MLC2 (Rabbit monoclonal (D18E2), 1:1000 for immunoblotting, Cell Signaling Technology, 8505)
MLCK (Rabbit monoclonal (EP1458Y), 1:1000 for immunoblotting, Abcam, ab76092)
OXPHOS (Rodent monoclonal (D18E2), 1:1000 for immunoblotting, Abcam, ab110413)
PDH (Rabbit monoclonal (C54G1), 1:1000 for immunoblotting, Cell Signaling Technology, 3205)
PDHK1 (Rabbit monoclonal (C47H1), 1:1000 for immunoblotting, Cell Signaling Technology, 3820)
LAMP1(Rabbit monoclonal (C54H11), 1:1000 for immunoblotting, Cell Signaling Technology, 3243)
LAMP2 (Rabbit monoclonal (C76H11), 1:200 for immunostaining, 1:1000 for immunoblotting, Proteintech,27823-1-AP)
CathepsinB (Rabbit monoclonal (D1C7Y), 1:200 for immunostaining, 1:1000 for immunoblotting, Cell Signaling Technology, 31718)
AMPK (Rabbit monoclonal (34.2), 1:1000 for immunoblotting, Abcam, ab80039)
p-AMPK (Rabbit monoclonal (40H9), 1:1000 for immunoblotting, Cell Signaling Technology, 2535)
AXIN (Rabbit monoclonal (C76H11), 1:1000 for immunoblotting, Cell Signaling Technology, 2087S)
Occludin (Rabbit monoclonal (EPR20992), 1:200 for immunostaining, 1:1000 for immunoblotting, Abcam, 216327)
Claudin-2 (Rabbit monoclonal (E1H9O), 1:1000 for immunoblotting, Cell Signaling Technology, 48120)

Claudin-4 (Rabbit monoclonal, 1:1000 for immunoblotting, Abcam, ab15104)
CathepsinL (Rabbit monoclonal, 1:1000 for immunoblotting, Abcam, ab203028)
CathepsinD (Rabbit monoclonal (EPR3057Y), 1:1000 for immunoblotting, Abcam, ab75852)
Caspase3 (Rabbit polyclonal, 1:1000 for immunoblotting, Proteintech, 19677-1-AP)
Caspase9 (Rabbit polyclonal, 1:1000 for immunoblotting, Proteintech, 10380-1-AP)
COXIV (Rabbit polyclonal, 1:1000 for immunoblotting, Proteintech, 11242-1-AP)
HRP-conjugated Affinipure Goat Anti-Mouse IgG(H+L), (1:5000 for immunoblotting, Proteintech, SA00001-1)
HRP-conjugated Affinipure Goat Anti-Rabbit IgG(H+L), (1:5000 for immunoblotting, Proteintech, SA00001-2)
Goat anti-Rabbit IgG (H+L) Highly Cross-Adsorbed Secondary Antibody, Alexa Fluor™ 488, (1:5000 for immunofluorescence, Thermo Fisher, A-11034)
Goat anti-Rabbit IgG (H+L) Highly Cross-Adsorbed Secondary Antibody, Alexa Fluor™ Plus 647, (1:5000 for immunofluorescence, Thermo Fisher, A32733)

Validation

All antibodies used have been validated in previous publications/by the manufacturer.
β-actin, Rabbit monoclonal [13E5], Cell Signaling Technology, 4970, https://www.cellsignal.cn/products/primary-antibodies/b-actin-13e5-rabbit-mab/4970?site-search-type=Products&N=4294956287&Ntt=4970&fromPage=plp&_requestid=1400315

ZO-1, Rabbit monoclonal [13E5], Thermo Fisher,61-7300, https://www.thermofisher.cn/cn/zh/antibody/product/ZO-1-Antibody-Polyclonal/61-7300

Claudin1, Rabbit polyclonal, Proteintech, 13050-1-AP, https://www.ptgcn.com/products/CLDN1-Antibody-13050-1-AP.htm
p-MLC2, Cell Signaling Technology, 3674, https://www.cellsignal.cn/products/primary-antibodies/phospho-myosin-light-chain-2-thr18-ser19-antibody/3674?site-search-type=Products&N=4294956287&Ntt=3674&fromPage=plp&_requestid=1401381

MLC2, Rabbit monoclonal (D18E2), Cell Signaling Technology, 8505, https://www.cellsignal.cn/products/primary-antibodies/myosin-light-chain-2-d18e2-rabbit-mab/8505

MLCK, Rabbit monoclonal (EP1458Y), Abcam, ab76092, https://www.abcam.cn/myosin-light-chain-kinasemlck-antibody-ep1458y-ab76092.html

OXPHOS, Rodent monoclonal (D18E2), Abcam, ab110413, https://www.abcam.cn/total-oxphos-rodent-wb-antibody-cocktail-ab110413.html

PDH, Rabbit monoclonal (C54G1), Cell Signaling Technology, 3205, https://www.cellsignal.cn/products/primary-antibodies/pyruvate-dehydrogenase-c54g1-rabbit-mab/3205?site-search-type=Products&N=4294956287&Ntt=3205&fromPage=plp&_requestid=1401673

PDHK1, Rabbit monoclonal (C47H1), Cell Signaling Technology, 3820, https://www.cellsignal.cn/products/primary-antibodies/pdhk1-c47h1-rabbit-mab/3820?site-search-type=Products&N=4294956287&Ntt=3820&fromPage=plp&_requestid=1401725

LAMP1, Rabbit monoclonal (C54H11), Cell Signaling Technology, 3243, https://www.cellsignal.cn/products/primary-antibodies/lamp1-c54h11-rabbit-mab/3243?site-search-type=Products&N=4294956287&Ntt=3243&fromPage=plp&_requestid=1401779

LAMP2, Rabbit monoclonal (C76H11), Proteintech, 27823-1-AP, https://www.ptgcn.com/products/LAMP2-Antibody-27823-1-AP.htm

CathepsinB, Rabbit monoclonal (D1C7Y), Cell Signaling Technology, 31718, https://www.cellsignal.cn/products/primary-antibodies/cathepsin-b-d1c7y-xp-rabbit-mab/31718?site-search-type=Products&N=4294956287&Ntt=31718&fromPage=plp&_requestid=1401833

AMPK, Rabbit monoclonal (34.2), Abcam, ab80039, https://www.abcam.cn/ampk-alpha-1--ampk-alpha-2-antibody-342-ab80039.html

p-AMPK, Rabbit monoclonal (40H9), Cell Signaling Technology, 2535, https://www.cellsignal.cn/products/primary-antibodies/phospho-ampka-thr172-40h9-rabbit-mab/2535?site-search-type=Products&N=4294956287&Ntt=2535&fromPage=plp&_requestid=1401904

AXIN, Rabbit monoclonal (C76H11), Cell Signaling Technology, 2087S, https://www.cellsignal.cn/products/primary-antibodies/axin1-c76h11-rabbit-mab/2087?site-search-type=Products&N=4294956287&Ntt=2087s&fromPage=plp&_requestid=1401951

Occludin, Rabbit monoclonal (EPR20992), Abcam, 216327, https://www.abcam.cn/occludin-antibody-epr20992-ab216327.html

Claudin-2, Rabbit monoclonal (E1H9O), Cell Signaling Technology, 48120, https://www.cellsignal.cn/products/primary-antibodies/claudin-2-e1h9o-rabbit-mab/48120?site-search-type=Products&N=4294956287&Ntt=48120&fromPage=plp&_requestid=1401981

Claudin-4, Rabbit monoclonal, Abcam, ab15104, https://www.abcam.cn/claudin-4-antibody-ab15104.html

CathepsinL, Rabbit monoclonal, Abcam, ab203028, https://www.abcam.cn/cathepsin-lmep-antibody-ab203028.html

CathepsinD, Rabbit monoclonal (EPR3057Y), Abcam, ab75852, https://www.abcam.cn/cathepsin-d-antibody-epr3057y-ab75852.html

Caspase3, Rabbit polyclonal, Proteintech, 19677-1-AP, https://www.ptgcn.com/products/CASP3-Antibody-19677-1-AP.htm

Caspase9, Rabbit polyclonal, Proteintech, 10380-1-AP, https://www.ptgcn.com/products/CASP9-Antibody-10380-1-AP.htm

COXIV, Rabbit polyclonal, Proteintech, 11242-1-AP, https://www.ptgcn.com/products/COX4I1-Antibody-11242-1-AP.htm

HRP-conjugated Affinipure Goat Anti-Mouse IgG(H+L), Proteintech, SA00001-1, https://www.ptgcn.com/products/HRP-conjugated-Affinipure-Goat-Anti-Mouse-IgG-H-L-secondary-antibody.htm

HRP-conjugated Affinipure Goat Anti-Rabbit IgG(H+L), Proteintech, SA00001-2, https://www.ptgcn.com/products/HRP-conjugated-Affinipure-Goat-Anti-Rabbit-IgG-H-L-secondary-antibody.htm

Goat anti-Rabbit IgG (H+L) Highly Cross-Adsorbed Secondary Antibody, Alexa Fluor™ 488, Thermo Fisher, A-11034, https://www.thermofisher.cn/cn/zh/antibody/product/Goat-anti-Rabbit-IgG-H-L-Highly-Cross-Adsorbed-Secondary-Antibody-Polyclonal/A-11034

Goat anti-Rabbit IgG (H+L) Highly Cross-Adsorbed Secondary Antibody, Alexa Fluor™ Plus 647, Thermo Fisher, A32733, https://www.thermofisher.cn/cn/zh/antibody/product/Goat-anti-Rabbit-IgG-H-L-Highly-Cross-Adsorbed-Secondary-Antibody-Polyclonal/A32733

Eukaryotic cell lines

Policy information about cell lines

Cell line source(s)	NCM460 cells are acquired from American Type Culture Collection (ATCC).
Authentication	The main test for authenticating NCM460 cells is short tandem repeat (STR) profiling, the analysis of microsatellite regions of DNA that have variable numbers of repeats and are located throughout the genome. PCR amplicons are generated using primers for regions that flank these polymorphic sites. After amplification, PCR products are resolved using capillary electrophoresis, and a profile for these repetitive regions is created.
Mycoplasma contamination	I confirmed that NCM460 cells tested negative for mycoplasma contamination.
Commonly misidentified lines (See ICLAC register)	There were no misidentified cells in our studies.

Animals and other organisms

Policy information about studies involving animals; ARRIVE guidelines recommended for reporting animal research

Laboratory animals	Fifteen-week old male (or female) IL-10-deficient (IL-10−/−) mice were obtained from the Shanghai Research Center for Model Organisms (Shanghai, China). Eight-week old male (or female) WT C57BL/6J mice were purchased from the Animal Institute of Southern Medical University (Guangzhou, China). Housing condition for mice: 20 ± 2 °C, 50 ± 5% humidity, 12h-12h light-dark cycles.
Wild animals	This study did not involve wild animals.
Field-collected samples	This study did not involve samples collected from the field.
Ethics oversight	All animal procedures were approved by the welfare and Ethical Committee for Experimental Animal Care of Southern Medical University.

Note that full information on the approval of the study protocol must also be provided in the manuscript.

Human research participants

Policy information about studies involving human research participants

Population characteristics	Demographics of all patients in the study were provided in Supplementary Table 1.
Recruitment	Serum were collected from patients the first time they are diagnosed or from healthy volunteers during physical examination.
Ethics oversight	The study was reviewed and approved by Medical Ethics Committee of Nanfang hospital.

Note that full information on the approval of the study protocol must also be provided in the manuscript.

Flow Cytometry

Plots

Confirm that:

- [x] The axis labels state the marker and fluorochrome used (e.g. CD4-FITC).
- [x] The axis scales are clearly visible. Include numbers along axes only for bottom left plot of group (a 'group' is an analysis of identical markers).
- [x] All plots are contour plots with outliers or pseudocolor plots.
- [x] A numerical value for number of cells or percentage (with statistics) is provided.

Methodology

Sample preparation	NCM460 cells were washed with PBS and subsequently incubated for 5 min at room temperature in the dark in 500 µl of 1× binding buffer containing 5 µl of Annexin V-APC and 10 µl of 7-AAD.
Instrument	Dakewe EXFLOW-206
Software	FlowJo 7.6.1 was used for data analysis.
Cell population abundance	No cell sorting was performed.
Gating strategy	Gating strategy is reporting in the Supplementary Fig 5, 10, 12. The scatter plot was generated by using SSC vs. FSC parameters and SSC(height) versus SSC(area) gating was applied to exclude doublets for analysis. Single NCM460 cells were gated with 7-AAD and Annexin V. 7-AAD and Annexin V negative cells were live cells; 7-AAD high and Annexin V high were late apoptosis or necrotic cells; 7-AAD low and Annexin V high were early apoptosis cells.

- [x] Tick this box to confirm that a figure exemplifying the gating strategy is provided in the Supplementary Information.
